# CoMutPlotter: a web tool for visual summary of mutations in cancer cohorts

**DOI:** 10.1186/s12920-019-0510-y

**Published:** 2019-07-11

**Authors:** Po-Jung Huang, Hou-Hsien Lin, Chi-Ching Lee, Ling-Ya Chiu, Shao-Min Wu, Yuan-Ming Yeh, Petrus Tang, Cheng-Hsun Chiu, Ping-Chiang Lyu, Pei-Chien Tsai

**Affiliations:** 1 Department of Biomedical Sciences, Chang Gung University, Taoyuan, Taiwan; 2 Graduate Institute of Biomedical Sciences, College of Medicine, Chang Gung University, Taoyuan, Taiwan; 3 Genomic Medicine Core Laboratory, Chang Gung Memorial Hospital, Linkou, Taiwan; 4 Institute of Bioinformatics and Structural Biology, National Tsing Hua University, Hsinchu, Taiwan; 5 Department and Graduate Institute of Computer Science and Information Engineering, Chang Gung University, Taoyuan, Taiwan

**Keywords:** Cancer mutational profile mutational signature TCGA

## Abstract

**Background:**

CoMut plot is widely used in cancer research publications as a visual summary of mutational landscapes in cancer cohorts. This summary plot can inspect gene mutation rate and sample mutation burden with their relevant clinical details, which is a common first step for analyzing the recurrence and co-occurrence of gene mutations across samples. The cBioPortal and iCoMut are two web-based tools that allow users to create intricate visualizations from pre-loaded TCGA and ICGC data. For custom data analysis, only limited command-line packages are available now, making the production of CoMut plots difficult to achieve, especially for researchers without advanced bioinformatics skills. To address the needs for custom data and TCGA/ICGC data comparison, we have created CoMutPlotter, a web-based tool for the production of publication-quality graphs in an easy-of-use and automatic manner.

**Results:**

We introduce a web-based tool named CoMutPlotter to lower the barriers between complex cancer genomic data and researchers, providing intuitive access to mutational profiles from TCGA/ICGC projects as well as custom cohort studies. A wide variety of file formats are supported by CoMutPlotter to translate cancer mutation profiles into biological insights and clinical applications, which include Mutation Annotation Format (MAF), Tab-separated values (TSV) and Variant Call Format (VCF) files.

**Conclusions:**

In summary, CoMutPlotter is the first tool of its kind that supports VCF file, the most widely used file format, as its input material. CoMutPlotter also provides the most-wanted function for comparing mutation patterns between custom cohort and TCGA/ICGC project. Contributions of COSMIC mutational signatures in individual samples are also included in the summary plot, which is a unique feature of our tool.

CoMutPlotter is freely available at http://tardis.cgu.edu.tw/comutplotter.

## Background

With the rapid evolution of next-generation technologies (NGS) combined with dropping costs, whole-exome sequencing (WES) has become a widely-accepted application for clinical research and diagnostic purposes. In the past few years, over 10,000 exomes across 40 distinct types of human cancer were generated by The Cancer Genome Atlas (TCGA) and the International Cancer Genome Consortium (ICGC). The Broad institute has released the GATK Best Practice workflow tailored to somatic variant discovery. Researchers can follow this standardize analysis protocol, making their results comparable to TCGA/ICGC projects. Variant annotation is a relatively mature and feasible work because of the state-of-the-art packages like ANNOVAR [1], VEP [2], SnpEff [3], and Oncotator [4]. However, an intuitive and convenient way for visualizing and interpreting genomic data from high-throughput technologies continues to be challenging. Inconsistent file formats used in handling mutation profiles may introduce additional problems in subsequent data integration, visualization and comparison.

CoMut plot [5–7] is widely used in cancer research publications as a visual summary of mutational landscapes in cancer cohorts. This summary plot can inspect gene mutation rate and sample mutation burden with their relevant clinical details, which is a common first step for analyzing the recurrence and co-occurrence of gene mutations across samples. There are two web -based applications, the cBioPortal [8] and iCoMut (http://firebrowse.org/iCoMut/), which allow users to create intricate visualizations from pre-loaded TCGA data. For custom data analysis, only certain file formats such as MAF and TSV format are supported at this stage, which are based on command-line packages [6, 7], making the production of customizable plots difficult to achieve, especially for non-bioinformatics researchers.

To address the needs for custom data and TCGA/ICGC data comparison, we have created CoMutPlotter, a web-based tool, for the production of publication quality graphs and to translate cancer mutation profiles into biological insights and clinical applications. A wide variety of file formats are supported by CoMutPlotter, which include Mutation Annotation Format (MAF), Tab-separated values (TSV) and Variant Call Format (VCF) files. It is worth noting that CoMutPlotter is the first tool of its kind that directly supports VCFs, a dominant output format of all variant discovery pipelines like the GATK Toolkit [9], VarScan [10], and SAMtools [11]. Deciphering signatures of the mutational processes in human cancer is a new trend in cancer research community [12–14] because these signatures are footprints of molecular aberrations occurring in tumors. Alexandrov et al. identified a list of 30 reference signatures and about half of these signatures can be attributed to endogenous processes such as enzymatic activity of DNA cytidine deaminases (AID/APOBEC), the deficiency of DNA mismatch repair, or mutations in *POLE* and to exogenous mutagens like tobacco, ultraviolet light and toxic chemicals [15].

Our specific aim to construct CoMutPlotter is to lower the barriers between complex cancer genomic data and researchers. In addition to specifying the mutation burden and types of individual samples, we also allow the user to plot clinical features with their respective samples, providing intuitive access to mutational profiles from TCGA/ICGC as well as custom cohort studies alongside their clinical attributes. CoMutPlotter also provides the most-wanted function for comparing mutational landscapes between custom cohort and TCGA/ICGC project. To gain insight into the mutational processes that have altered the cancer genome, contributions of COSMIC signatures are quantified at sample resolution and integrated in the summary plot as dot matrix, which is a unique feature of CoMutPlotter. CoMutPlotter is freely available at http://tardis.cgu.edu.tw/comutplotter.

## Implementation

### CoMutPlotter framework

**Fig. 1:**
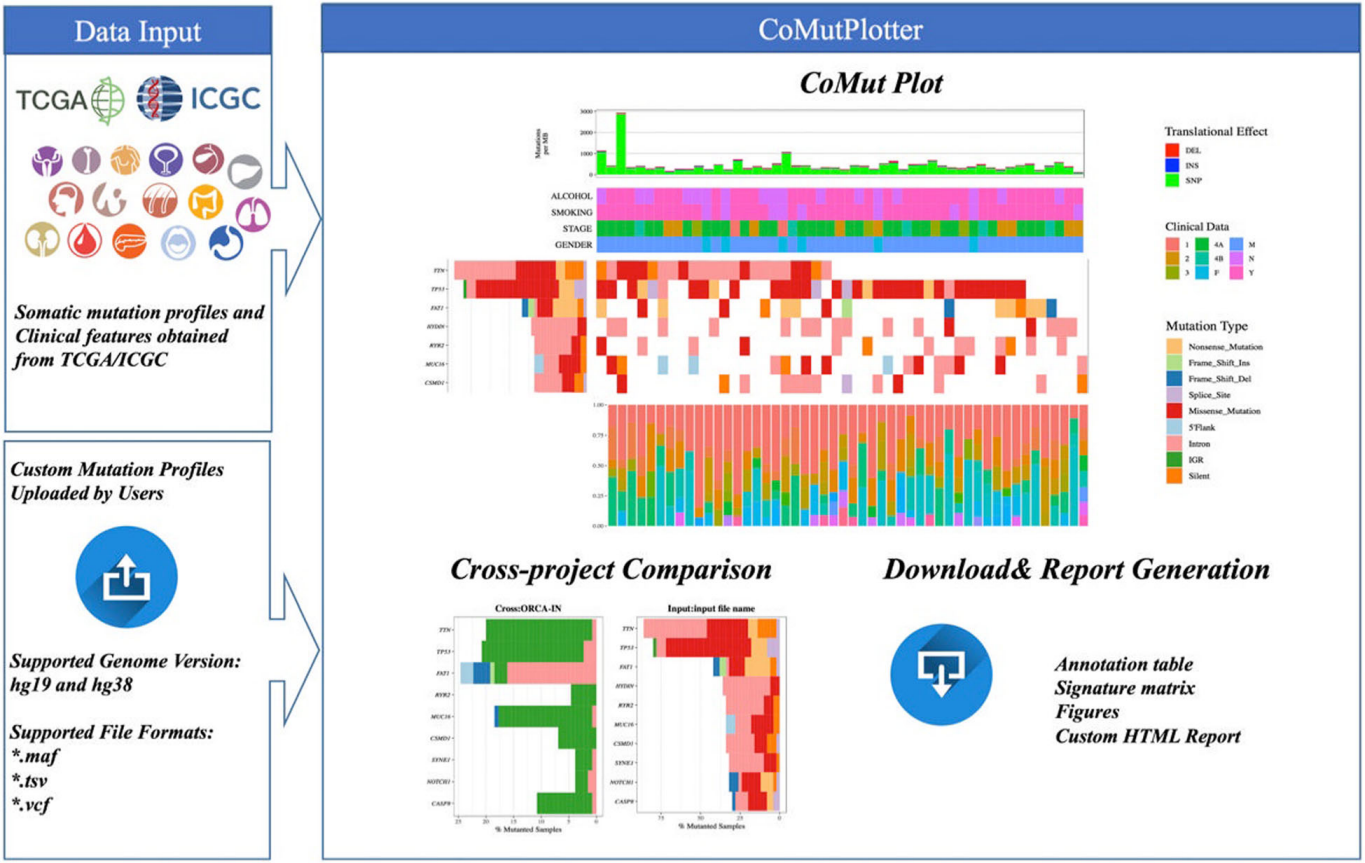
Framework of CoMutPlotter. In addition to TCGA/ICGC data, CoMutPlotter can take mutational profiles from custom projects in MAF, TSV and VCF formats. CoMutPlotter consists of three major parts: 1) Data input, 2) pre-loaded TCGA/ICGC database, and 3) Output. The output part can be further divided into three panels, which includes the “CoMut Plot” panel, the “Cross-project comparison” panel and the “Download & Report Generation” panel

CoMutPlotter provides an intuitive web interface to receive mutation profiles obtained from cancer sequencing projects. Mutation Annotation Format (MAF) is widely used in TCGA cancer studies for storing mutation profiles, which is also the basis for many downstream analyses such as variant annotation, driver gene detection, mutual exclusivity analysis, and mutational signature identification. In addition to MAF file, CoMutPlotter also includes function to convert ICGC tab-separated values (TSV) file and standard Variant Call Format (VCF) file to MAF file, making this tool more accessible to wider researchers. CoMutPlotter not only provides complete functions for performing analyses mentioned above but also creates an interactively framework to present and summarize the important characteristics of the multidimensional analysis results from a custom cancer cohort. For the convenience of comparative analysis between custom data and TCGA/ICGA data, 73 mutation profiles were downloaded from TCGA and ICGC Data Portal and compiled as pre-loaded database. The PHP and R script are used to summarize all the generated results into an integrative plot to grasp the global characteristics of a mutation profile and to reveal the co-occurrence of mutations and samples. Download links are also provided to download publication-quality figures, significantly mutated gene list and detailed annotation table (Fig. 1).

### Data input

CoMutPlotter accepts three dominant formats of mutation profiles, including MAF, TSV, and VCF formats. To make data management and analysis more efficient, mutation profiles in diverse formats are converted to MAF format before entering subsequent analyses. A custom script for file format conversion is available for download (http://tardis.cgu.edu.tw/comutplotter/comutplotter_tutorial/implementation.html#for-custom-study-with-large-number-of-vcf-files) when users try to deal with a study cohort with large number of VCF files. To perform in-depth comparisons between clinical features or study designs within a cancer cohort, the demographic profile can also be uploaded along with the mutation profiles. Detailed instructions on the usage of the custom script and the acceptable format of the demographic file can be found on the tutorial page (http://tardis.cgu.edu.tw/comutplotter/Tutorial/comutTutorial.html#2_data_input).

### Functional consequence annotation

Functional annotation of variants is a key step [16] in the analysis of cancer sequencing data, and annotation results may have a substantial influence on the final conclusions of cohort studies. Despite using the same transcript sets (e.g., REFSEQ or ENSEMBL) as the basis for annotation, there still have about 20% disagreement between annotation results generated from well-recognized methodologies such as ANNOVAR, SnpEff, and Variant Effect Predictor. To capture the expected variant annotations in concordance with TCGA published cancer studies, GENCODE release 19 was used to construct cancer-relevant transcripts as instructed by previous study (https://www.broadinstitute.org/~lichtens/oncobeta/tx_exact_uniprot_matches.AKT1_CRLF2_FGFR1.txt) [4]. The local installed version of Broad’s Oncotator [4] was used to perform the annotation tasks, making functional annotation of variants become a reproducible step and ensuring the annotation results are comparable between custom cohort and TCGA/ICGC studies. The mutation rates of synonymous and non-synonymous variants can be calculated in individual samples, which are subsequently rendered into a stacked bar chart for monitoring selective pressure acting on protein-coding genes. Gene mutations can be further classified into missense, nonsense, stop-gain, insertion and deletion, frameshift and splice site mutations, depending on where they occur and whether they alter the composition of proteins.

### Cancer driver gene identification

International cancer projects are underway through The Cancer Genome Atlas (TCGA) and the International Cancer Genome Consortium (ICGC) aim to establish a comprehensive catalogue of cancer associated genes across all cancer types. However, most of the existing analytical methods fail to account for mutational heterogeneity that affects the background mutation rate and may led to the identification of many specious genes. Lawrence et al. has developed a new method, named MutSigCV [17], to address the issue of mutational heterogeneity, which is correlated with transcriptional activity, DNA replication timing, and mutation frequency variability across patients. To facilitate the identification of genes truly associated with cancer and to make driver gene detection more accessible to users, CoMutPlotter has incorporated MutSigCV as a critical analysis module. The mutation profiles uploaded by users are converted to MAF format as mentioned above and then subjected to MutSigCV to determine significantly mutated genes with false discovery rates (q-value) less than or equal to 0.1. Since the mutation profiles of 73 cancer projects have been downloaded from TCGA/ICGC Data Portal, we also applied the MutSigCV method to identify diver genes in individual cancer projects. Based on the pre-calculated results, users can easily compare the resulting gene lists between custom study cohort and published cancer projects.

### Mutational signature recognition

Mutational signatures are patterns of somatic mutations hidden in cancer genomes, which can be represented as different combinations of 96 available trinucleotide mutation contexts. Each mutational signature may be associated with specific kinds of mutational processes resulting from exogenous and endogenous mutagens such as ultraviolet radiations, tobacco-related exposures and abnormal activity of enzymes. Up to date, 30 distinct mutational signatures have been identified and categorized in COSMIC database using the WTSI Mutational Signature Analysis Framework [12]. However, large cohorts and sufficient computing resources are required by existing analysis framework of WTSI. Moreover, quantifying known signatures in individual samples is not possible under the current WTSI framework when sample sizes are small. For known signatures identification and quantification, the R deconstructSigs package [18] was used to determine the composition of mutational signatures in individual tumor samples. A dot matrix plot is used to show the percentage contribution of the identified signatures in each sample. The proposed etiology of each signature can be downloaded as a summary table, which may be beneficial to explore different combinations of mutational signatures that are representative in distinct groups of patients, to depict potential therapeutic targets and to reveal new connections between mutational processes and clinical features.

### Report generation

**Fig. 2:**
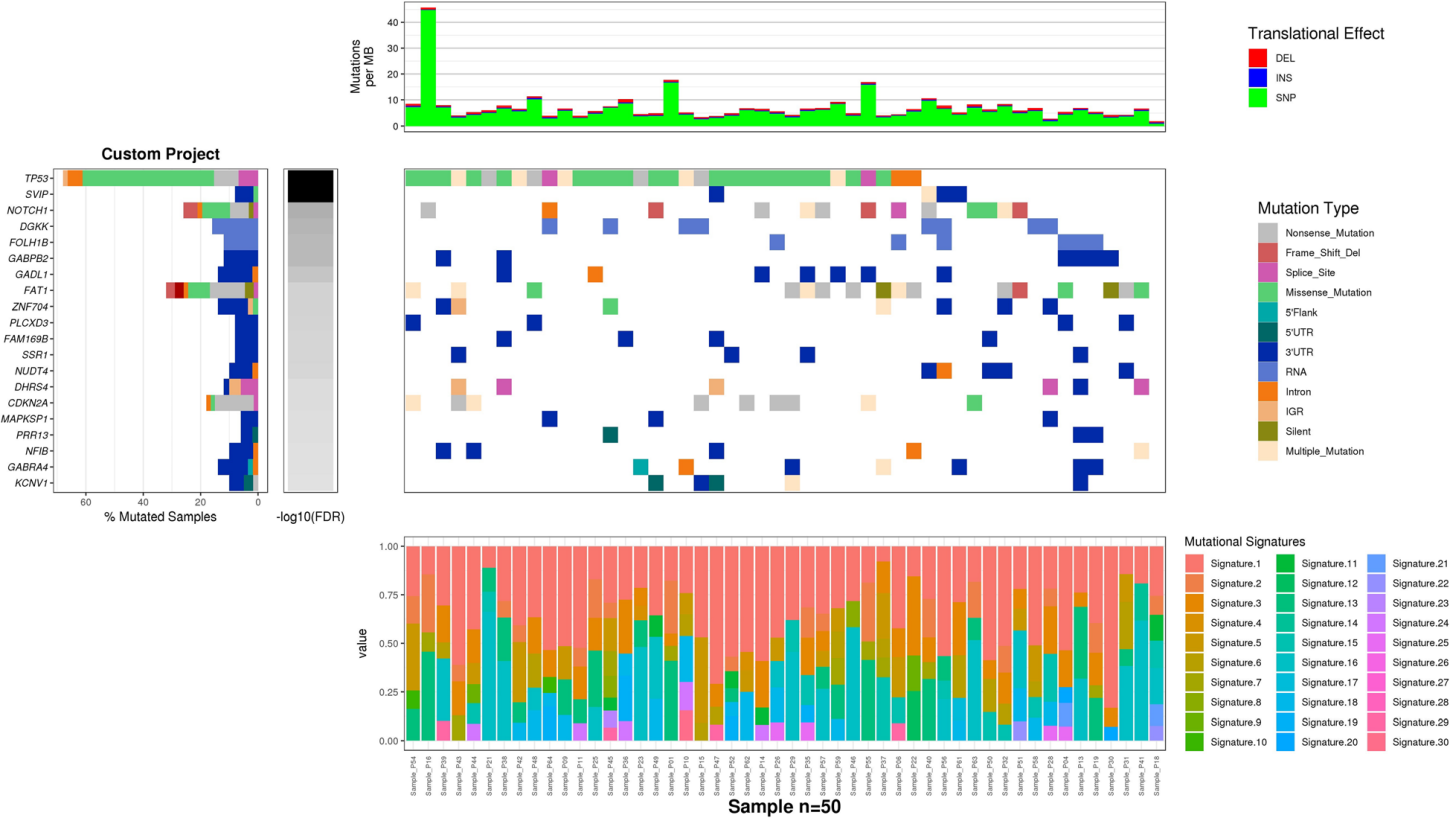
Output of CoMutPlotter. The mutation profile of custom cohort is displayed as a heatmap in the main body of the plot, using different glyphs and colors to reveal diverse type of gene mutations in different patients. The significantly mutated genes identified by MutSigCV are displayed as bar chart along the left axis of the plot, ordered according to negative q-values in log transformations, which can also be ordered according to gene mutation frequencies. Clinical information is also displayed as heatmap. At the lower portion of the plot, contributions of 30 COSMIC mutational signatures are rendered as percentage stacked bar chart

With the improved completeness of software packages over the past few years, data analysis in cancer research has gradually become a feasible tack. Many state-of-the-art analysis packages such as GATK [9], Oncotator [4] and MutSigCV [17] have been released by the Broad Institute and users can apply these packages to analysis their own data when computing power is not a concern. However, most of the existing packages lack a mechanism to create a visual summary for effectively communicating personal findings to research community, which can be the most import and challenging step of scientific research. As shown in Fig. 2, CoMutPlotter has summarized all the analysis results mentioned in above sections into a single integrative plot. The mutation profile of custom cohort is displayed as a heatmap in the main body of the plot, using different glyphs and colors to reveal diverse type of gene mutations in different patients. The significantly mutated genes identified by MutSigCV are displayed as bar chart along the right axis of the plot, ordered according to negative q-values in log transformations. Along the top axis is the density of mutations for each patient while the mutation frequency for each gene is rendered along the left axis. The clinical features can be retrieved from the uploaded demographic profile aligned according to the respective patients and rendered as a second heatmap on the top panel of the plot. Contributions of COSMIC mutational signatures in each patient are shown as dot matrix, rendered at the bottom panel of the plot. The dynamic framework of CoMutPlotter provides both sorting and filtering functions on the left panel. Users can sort the list of genes according to mutation frequencies or the FDR q-values. Filters are provided based on items such as custom gene list and mutation types, facilitating users to focus on their target of interest. A “report generation” button is provided to create a publication-quality figure, often seen in cancer research publications as a visual summary of genetic aberrations in cancer cohorts along with table with detailed annotation information.

## Results and discussion

### Example of use

As a proof-of-concept experiment, we applied CoMutPlotter to analyze our published datasets [17, 18], which contain 50 sets of whole-exome sequencing data from oral cancer patients in Taiwan. In this study cohort, 24,051 mutation events that correspond to 23,495 unique somatic mutations were identified and recorded in the MAF file, which can be downloaded through the following link: (http://tardis.cgu.edu.tw/comutplotter/oscc_50.maf.zip). For cross-project comparison, the same analysis workflow was also applied to analyze 178 sets of whole-exome sequencing data from oral cancer patients in India [19], which can be downloaded from the ICGC Data Portal through the following link: (https://dcc.icgc.org/api/v1/download?fn=/release_27/Projects/ORCA-IN/simple_somatic_mutation.open.ORCA-IN.tsv.gz).

Detailed exemplary outputs for 50 oral tumors can be found on the CoMutPlotter demonstration page at (http://tardis.cgu.edu.tw/comutplotter/Demo/). Detailed instructions can refer to the following link http://tardis.cgu.edu.tw/comutplotter/comutplotter_tutorial/example-of-use.html.

### Output summary

After the successful submission of a job, a dynamic progress bar will be displayed, indicating processing statuses such as job queuing, format conversion, variant annotation, significantly mutated gene identification, mutational signature decomposition, and CoMut plot generation. The standard output can be separated into three webpage panels, including CoMut plot, Cross-project comparison, and Download & Report generation.

In the “CoMut plot” panel, stacked bar graphs are used to represent the mutation burden of individual samples, the compositions of translational effects and the most frequently affected genes in a study cohort, rendered at the top and left-hand side of the resulting CoMut plot. In the main body of the plot, heat map is used to visualize multiple genomic alteration events in individual samples and to render diverse mutation types by different color schemes. Percentage stacked bar is used to represent the identified COSMIC signatures in each sample, which can be switched to dot matrix to better convey the contributions of respective mutational processes. The resulting plot can be ordered not only by gene mutation frequency but also significant FDR values calculated from MutSigCV algorithm. Moreover, users can create custom plot according to the function for mutation types selection and custom gene list.

In the “Cross-project comparison” panel, users can easily compare their study cohort to pre-loaded cancer projects from TCGA/ICGC. Despite that users can retrieve or create CoMut plot for each TCGA/ICGC project using on-line resources or command-line tools, only CoMutPlotter provides the function to render the comparison result in the same plot and in the same gene order, making cross-project comparison become an easy task. As shown in Fig. 2, users can easily depict the convergent and divergent gene mutation frequencies between Taiwan and India populations of the same cancer type.

In the “Download & Report generation” panel, detailed information about the significantly mutated genes, contributions of mutational signatures in individual samples and the resulting CoMut plot can be downloaded from our server as separated tables, figures or integrated HTML file.

### Comparison of the features across similar tools

Over the past few years, many packages have been developed to meet the needs for visual summary of mutations in cancer cohorts. These packages can be further classified into two groups. One group is web-based tools and the other group is command line tools. The cBioPortal and iCoMut are two representative packages of web-based tools and the benefit part is easy-to-use while the shortcoming is limited to the cancer projects from TCGA or ICGC. The command-line tools have their inherited problem, only support MAF format as their input format and likely limited to specific users with bioinformatics background. Furthermore, the issue of cross-project comparison has never been covered by existing packages as well as the functionality of mutational signature analysis. CoMutPlotter aims to provide the most comprehensive set of features to address all of these issues. A more detailed comparisons of similar existing software is summarized in Table 1.

**Table 1:** Comparison of the features of similar tools for CoMut-like plot generation

Software	CoMutPlotter	iCoMut^a^	cBioPortal^b^	GenVisR^c^	maftools^d^
Usage	Web	Web	Web	Command	Command
Supported file format	MAF TSV VCF	TCGA data	TCGA/ICGC data	MAF TSV	MAF
Significantly mutated gene identification	✓	✓	✗	✗	✗
Cross-project comparison	✓	✗	✗	✗	✗
Mutational signature analysis	✓	✗	✗	✗	✓

^a^ http://firebrowse.org/iCoMut/

^b^ http://www.cbioportal.org

^c^ https://bioconductor.org/packages/release/bioc/html/GenVisR.html

^d^ https://bioconductor.org/packages/release/bioc/html/maftools.html

### Future development

The planned future development of new features will be focused on incorporating the copy number variation and gene expression data into the resulting CoMut plot.

## Conclusions

CoMutPlotter is the first tool of its kind that supports VCF file, the most widely used file format, as its input material. CoMutPlotter provides the most complete solution staring from file format conversion all the way to variant annotation, driver gene identification, mutational signature recognition and CoMut plot generation. Moreover, CoMutPlotter also provides the most-wanted function for comparing mutation patterns between custom cohort and TCGA/ICGC project. Contributions of COSMIC mutational signatures in individual samples are also included in the summary plot, which is a unique feature of our tool.

CoMutPlotter is freely available at http://tardis.cgu.edu.tw/comutplotter.

## Availability and requirements

Project name: CoMutPlotter.

Project home page: http://tardis.cgu.edu.tw/comutplotter

Operating system(s): Platform independent.

Programming language(s): R, PHP, Shell Script and JavaScript.

Other requirements: Supported browsers Safari, Google Chrome, Firefox, Internet Explorer 11 and Microsoft Edge.

License: GNU GPL version 3.

Any restrictions to use by non-academics: none.

## Abbreviations

COSMIC: Catalogue of Somatic Mutations in Cancer
GATK: Genome Analysis Toolkit
ICGC: International Cancer Genome Consortium
MAF: Mutation Annotation Format
TCGA: The Cancer Genome Atlas
TSV: Tab-separated Values
VCF: Variant Call Format
WES: Whole-exome sequencing
